# Is the mode of childbirth delivery linked to the prevalence of early childhood caries? A systematic review and meta-analysis

**DOI:** 10.1007/s40368-021-00621-6

**Published:** 2021-05-10

**Authors:** K. Boustedt, J. Dahlgren, J. Roswall, S. Twetman

**Affiliations:** 1 Maxillofacial Unit, Halland Hospital, 30185 Halmstad, Sweden; 2 Department of Pediatrics, The Sahlgrenska Academy, University of Gothenburg, Gothenburg, Sweden; 3 Department of Pediatrics, Halland Hospital, Halmstad, Sweden; 4 Department of Odontology, Faculty of Health and Medical Sciences, University of Copenhagen, Copenhagen, Denmark

**Keywords:** Caesarean section, Caries, Children, Meta-analysis, Vaginal birth, Teeth

## Abstract

**Aim:**

The mode of childbirth delivery can influence the child’s future health and the aim of this study was to explore the association between the delivery mode and the prevalence of early childhood caries.

**Methods:**

We searched the PubMed, Google Scholar and Cochrane databases up to September 15, 2020. Two independent reviewers screened the papers for relevance, extracted data and assessed the risk of bias with the Newcastle–Ottawa Scale. We performed a random effects meta-analysis to pool the prevalence of early childhood caries according to the mode of delivery.

**Results:**

The authors included 11 studies in the review, comprising 47,688 children with vaginal delivery and 10,994 with caesarean section (C-section). The publication years ranged from 1997 to 2020 and included birth cohorts, cross-sectional, register-based and case–control studies. We assessed three publications with low or moderate risk of bias. The median caries prevalence in the C-section group was 56.4% compared to 45.9% in the vaginal group and this difference was statistically significant (*p* < 0.05). The pooled overall odds ratio was 1.48 (95% CI 1.07–2.05) indicating a weak but statistically significant trend towards a higher caries occurrence among children delivered with C-section. The certainty of this finding was low due to heterogeneity and inconsistencies across the studies.

**Conclusion:**

We found a weak but inconsistent association between the mode of delivery and the prevalence of early childhood caries. Further studies based on representative, prospective cohorts reporting a standardized core outcome set are required to answer the research question with higher certainty.

**Supplementary Information:**

The online version contains supplementary material available at 10.1007/s40368-021-00621-6.

## Introduction

Early childhood caries (ECC) is defined as the presence of one or more decayed, missed or filled surface in any primary tooth of a child under 6 years of age (Tinanoff et al. 2019). It is a common condition and more than 600 million children worldwide are estimated to suffer from untreated caries in their deciduous teeth (Marcenes et al. 2013). The severe forms of ECC may cause difficulties to eat, sleep and attaining school due to loss of tooth substance and pain (Anil and Anand 2017; Pitts et al. 2017). A systematic review has reported more than 100 unique biological, genetic, social and behavioural risk factors for early childhood caries in a complex interplay (Kirthiga et al. 2019). Among them, maternal and perinatal risk factors, such as preterm birth and gestational age, have been investigated in relation to ECC with mixed results (Boustedt et al. 2020; Occhi-Alexandre et al. 2020; Twetman et al. 2020). In this context, the mode of delivery is of particular interest, since the frequency of caesarean section (C-section) on maternal request has increased unprecedented over the recent decades (Begum et al. 2020) and it is important to have solid information if this may affect the future oral health of the child. To our knowledge, the association between the mode of delivery and ECC has previously been addressed in one systematic review (Antão et al. 2019). Based on four studies, the authors found no consistent relationship between the birth mode and the caries risk. However, the search for relevant literature was limited up to year 2015, and consequently, we found it of interest to update this information. The aim of this study was to systematically examine and pool the available literature on the mode of delivery and caries prevalence in the primary dentition. The focused question was “Is there an association between the mode of delivery (C-section vs. vaginal delivery) and the prevalence of early childhood caries?”.

## Methods

We conducted this systematic review according to a predefined plan and followed the PRISMA statement (Moher et al. 2009). The PECO was: *Population*—Toddlers and preschool children, 2–6 years of age; *Exposure*—Delivery through caesarean section (CS); *Comparison*—Vaginal delivery (V); *Outcome—*Prevalence of early childhood caries in the primary dentition up to 6 years of age. The authors included prospective studies (birth cohorts), cross-sectional studies, case–control designs and register-based studies. Articles reporting convenience samples, case series and case reports were excluded. The study protocol was not preregistered in a publicly assessable database.

### Search methods for identification of studies

The following electronic databases were searched from inception up to 15 September 2020: PubMed, Google Scholar and the Cochrane Oral Health Group’s Trials Register. The search words used were [(preschool child OR infant) AND (mode of delivery OR caesarean section OR C-section OR vaginal birth OR childbirth OR risk factors) AND (dental caries OR early childhood caries OR tooth decay)]. Only original peer-reviewed studies published with at least full abstracts and tables in English were eligible for inclusion. We excluded narrative reviews and grey literature, such as textbooks, conference papers, monographs and thesis. Clinical trials.gov was used to identify registered ongoing studies by combining the phrase “delivery mode” with “dental caries”. The reference lists of all identified studies, including one systematic review, were hand-searched for additional studies.

### Selection of studies

**Fig. 1:**
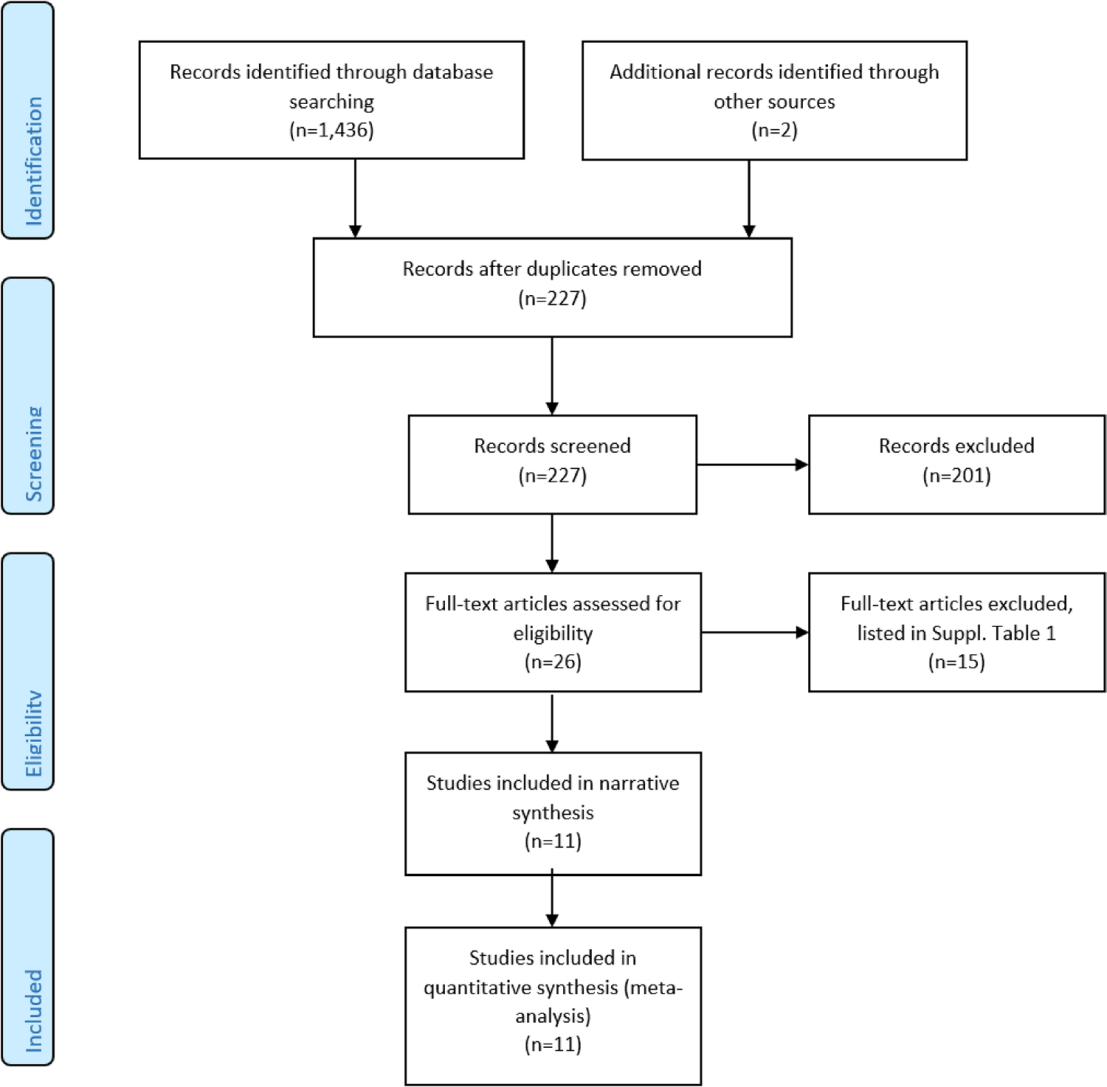
Flow chart for search, screening and exclusion of literature

Two authors (ST, KB) assessed the titles and abstracts of potentially eligible studies independently. If there was any doubt, the authors ordered the full-text papers for further evaluation. Any disagreement was resolved by discussions with a third author (JD). A flowchart of the study selection is shown in Fig. 1. The excluded studies and the main reason for their exclusion are listed in supplementary Table S1.

### Data extraction and management

Two examiners (ST, KB) read the full papers and extracted data independently. The authors tabulated the following data: first author, year of publication, country of origin, study design, sample size, follow-up age, method and level of caries scoring, the number of examiners, calibration of examiners and sample size calculation. The outcome measure was prevalence of caries (decayed, missed and filled primary teeth > 0, and/or non-cavitated enamel lesions > 0) on subject level, expressed as percent. In studies, where data were unclear or missing, we contacted the corresponding author by e-mail for clarifications. If authors failed to provide additional data after two reminders, the publication was excluded.

### Assessment of risk of bias in included studies

Each study was independently examined for the risk of bias by two authors (ST, KB). Any disagreement was discussed within the whole author group. We used the Newcastle–Ottawa Scale (NOS) to assess the quality of the included case–control, cohort and cross-sectional studies as suggested by Wells and co-workers (2014). We omitted item number four under the selection domain (“Demonstration that outcome of interest was not present at start of study”) and the maximum score was, therefore, eight stars. An overall assessment of the risk of bias was made for each included study: ≥ 7 stars = low risk of bias, 6 stars = moderate risk of bias, and ≤ 5 stars = high risk of bias. The risk of bias across the included studies was assessed with the GRADE approach (Guyatt et al. 2011).

### Data synthesis

The authors conducted a narrative synthesis of the included studies and made a Chi-squared test to compare the medium prevalence of caries between the two birth groups. We applied unadjusted dichotomous data (caries vs. caries-free) to calculate the odds ratio with 95% confidence intervals for each separate study. The caries prevalence according to mode of delivery was then pooled in a random effects model using the Review Manager 5.3 tool (The Nordic Cochrane Centre, Copenhagen, Denmark). The clinical heterogeneity was assessed by examining the characteristics of the studies, the similarity between the types of participants and the outcomes of the included studies in this review.

## Results

### Characteristics of the included studies

The authors included 11 studies covering 58,662 children in this review of which 47,688 had a vaginal delivery and 10,994 were born through C-section. The main characteristics are listed in Table 1. Six studies were cross-sectional trials (Zhou et al. 2011; Garcia-Castro and Perona-Miguel de Priego 2017; Shanthosh Raj et al. 2018; Pattanaporn et al. 2018; Sridevi et al. 2018; Korolenkova et al. 2020), two utilized population registers (Barfod et al. 2012; Brandquist et al. 2017), one was based on a prospective birth cohort (Boustedt et al. 2018) and two had a case–control design (Peretz and Kafka 1997; Nakai et al. 2016). The number of participating children per study ranged from 100 to 55,092 with a median value of 352. Four studies were from Europe (Barfod et al. 2012; Brandquist et al. 2017; Boustedt et al. 2018; Korolenkova et al. 2020), five from Asia (Zhou et al. 2011; Pattanaporn et al 2013; Nakai et al. 2016; Sridevi et al. 2018; Shanthosh Raj et al. 2018), one from South America (Garcia-Castro and Perona-Miguel de Priego 2017) and one from Israel (Peretz and Kafka 1997). The publication years ranged from 1997 to 2020. The threshold for caries detection was based on dentin (cavitated) level in 10 studies and only one scored non-cavitated early enamel lesions in addition to cavities (Boustedt et al. 2018). In general, bitewing radiographs were not used, except for one study, in which radiographs were captured based on individual indications (Boustedt et al. 2018). The number of clinical examiners ranged from one to six but few of the included publications reported data on examiner calibration and reliability tests. Caries status was scored at different ages, ranging from 8 months up to 6 years of age.

**Table 1:** Main characteristics of the included studies

First author, year	Country	Type	CS/V (*n*)	Age	Caries level^a^	Examiners^b^
Barfod, 2012	Denmark	Registry	151/443	3 years	deft	NA^c^/NA/NA
Boustedt, 2018	Sweden	Prospective	107/184	5 years	deft + enamel	2/yes/no
Brandquist, 2017	Sweden	Registry	9,587/45,505	3 years	deft	NA/NA/NA
García-Castro, 2017	Peru	Cross-sectional	52/73	2–5 years	deft	?/?/NR^d^
Korolenkova, 2020	Russia	Cross-sectional	52/113	3–6 years	deft	NR/NR/NR
Nakai, 2016	Japan	Case–control	42/113	3 years	deft	NR/NR/yes
Pattanaporn, 2013	Thailand	Cross-sectional	166/186	3–5 years	deft	2/no/no
Peretz, 1997	Israel	Case–control	37/63	3–4 years	BBTD	NR/NR/NR
Shantosh Raj, 2018	India	Cross-sectional	369/351	8 months–6 years	deft	1/NR/NR
Sridevi, 2018	India	Cross-sectional	264/426	3–6 years	deft	1/yes/yes
Zhou, 2011	China	Cross-sectional	163/231	2 years	deft	1/NR/yes

^a^*deft* decayed (cavitated lesions), extracted filled teeth, *enamel* initial non-cavitated lesions; *BBTD* carious involvement of at least three maxillary incisors on the buccal surface, irrespective of severity of the lesions

^b^Number of examiners/calibrated (yes or no)/sample size calculation (yes or no)

^c^NA = not applicable

^d^NR = not reported

### Quality assessment

The risk of bias is shown in Table 2. One of the included studies had a low risk of bias (Brandquist et al. 2017), two displayed a moderate risk (Zhou et al. 2011; Boustedt et al. 2018) and eight had a high risk of bias (Peretz and Kafka 1997; Barfod et al. 2012; Pattanaporn et al. 2013; Nakai et al. 2016; Garcia-Castro and Perona-Miguel de Priego 2017; Sridevi et al. 2018; Shanthosh Raj et al. 2018; Korolenkova et al. 2020). All studies except three scored at least one star in each of the three domains of “selection”, “comparability” and “outcome”. The main weaknesses were lack of representativeness, small sample sizes and outcome assessments. The GRADE certainty rating across the studies was very low.

**Table 2:** Risk of bias according to the Newcastle–Ottawa Scale

Prospective Cohort Studies	Selection				Comparability	Outcome/Exposure			Total Score
	1	2	3	4	5	6	7	8	
Barfod (2012)	x	x	★	NA	★	x	★	★	4
Boustedt (2018)	x	★	★	NA	★	★	★	★	6
Brandquist (2017)	★	★	★	NA	★★	x	★	★	7
Garcia-Castro (2017)	x	x	★	NA	★	★	x	★	4
Korolenkova (2020)	x	x	★	NA	★	x	★	★	5
Nakai (2016)	x	x	★	NA	x	★	★	★	4
Pattanaporn (2013)	x	x	★	NA	★	★	★	★	5
Peretz (1997)	x	x	★	NA	x	★	★	★	4
Shantosh Raj (2018)	x	x	★	NA	★	★	x	★	4
Sridevi (2018)	x	x	★	NA	x	★	★	★	4
Zhou (2011)	★	★	★	NA	★	★	x	★	6

*NA* denotes “not assessed”

Criteria for cohort and registry studies: (1) representativeness of the exposed cohort; (2) selection of the non-exposed cohort; (3) ascertainment of exposure; (4) demonstration that outcome of interest was not present at start of study; (5) comparability of cohorts on the basis of the design or analysis; (6) assessment of outcome; (7) was follow-up long enough for outcomes to occur; (8) adequacy of follow-up of cohorts

Criteria for case–control studies: (1) is the case definition adequate?; (2) representativeness of the cases; (3) selection of controls; (4) definition of controls; (5) comparability of cases and controls on the basis of the design or analysis; (6) ascertainment of exposure; (7) same method of ascertainment for cases and controls; (8) non-response rate

### Early childhood caries

**Fig. 2:**
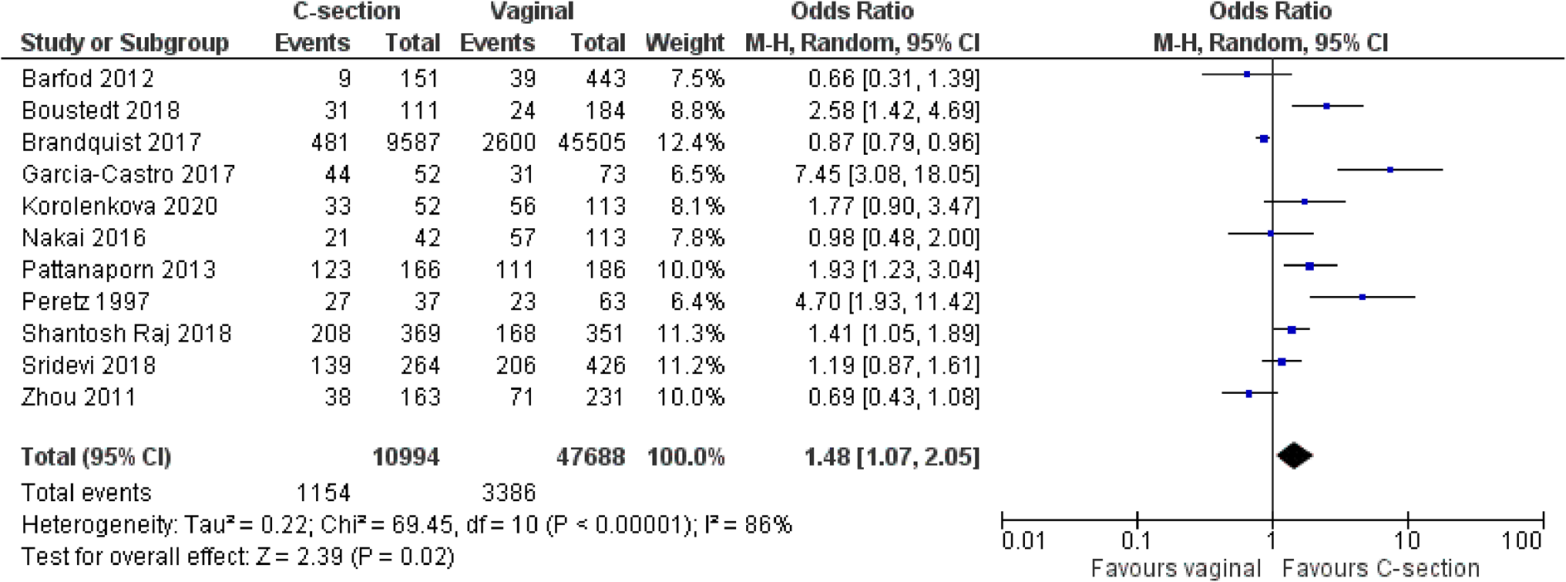
Pooled odds ratio for early childhood caries in relation to the mode of delivery

The prevalence of early childhood caries ranged from 5.3% to 84.6% (median 56.4%) in the C-section group and from 6.1% to 59.6% (mean 45.9%) in the vaginal group. This difference in proportions was statistically significant (*p* < 0.05). The crude univariate odds ratio (OR) for the separate studies is shown in Table 3. There was a considerable inconsistency between the studies. The prevalence of ECC was significantly higher (*p* < 0.05) among the C-section children in five studies (Peretz and Kafka 1997; Pattanaporn et al. 2013; Garcia-Castro and Perona-Miguel de Priego 2017; Boustedt et al. 2018; Shanthosh Raj et al. 2018) and four reported no significant difference between the two modes of delivery (Barfod et al. 2012; Zhou et al. 2013; Sridevi et al. 2018; Korolenkova et al. 2020). One study found significantly more caries in the vaginal group (Brandquist et al. 2017) but the caries prevalence was very low in both groups. When the studies were pooled in a meta-analysis (Fig. 2), the overall OR was 1.48 (95% CI 1.07–2.05) indicating a weak but statistically significant trend of a higher prevalence of early childhood caries among children delivered with C-section. The heterogeneity was very high with an *I*^2^ value of 86% (Tau^2^ = 0.22).

**Table 3:** Prevalence of early childhood caries, expressed as percent, according to mode of delivery

First author year	C-section C/CF* (*n*); prevalence	Vaginal C/CF* (*n*); prevalence	OR (95% CI)	*p*
Barfod (2012)	9/142; 6.0%	39/404; 8.8%	0.66 (0.31–1.39)	NS
Boustedt (2018)	31/80; 29.0%	24/160; 13.0%	2.58 (1.42–4.69)	< 0.01
Brandquist (2017)	481/9,106; 5.3%	2,600/42,905; 6.1%	0.87 (0.79–0.96)	< 0.01
Garcia-Castro (2017)	44/8; 84.6%	31/42; 42.5%	7.45 (3.08–18.02)	< 0.001
Korolenkova (2020)	33/19; 62.8%	56/57; 49.6%	1.77 (0.90–3.47)	NS
Nakai (2016)	21/21; 50.0%	57/56; 50.4%	0.98 (0.48–2.00)	NS
Pattanaporn (2013)	123/43; 73.8%	111/75; 59.6%	1.93 (1.22–3.04)	< 0.01
Peretz (1997)	27/10; 73.0%	23/40; 36.5%	4.70 (1.93–11.42)	< 0.001
Shantosh Raj (2018)	208/161; 56.4%	168/183; 47.9%	1.41 (1.05–1.89)	< 0.05
Sridevi (2018)	139/125; 52.7%	206/220; 45.9%	1.19 (0.87–1.62)	NS
Zhou (2011)	38/125; 23.3%	71/160; 30.7%	0.69 (0.43–1.08)	NS

The odds ratio (OR) and 95% confidence interval (CI) are univariate and unadjusted values

*C/CF: caries (yes)/caries free; *NS* not statistically significant

## Discussion

The mode of delivery can influence the child’s future health with an increased risk of asthma and obesity when delivered with C-section (Keag et al. 2018). Caesarean delivery affects the biodiversity of the gut microbiota in infants (Bäckhed et al. 2015) and similar observations are available from the oral and salivary microbiome (Nelun Barfod et al. 2011; Boustedt et al. 2015; Li et al. 2018). Infants born by C-section seem initially to have a less diverse salivary microbiota and harbour fewer beneficial health-associated species bacteria compared to children with vaginal birth, albeit a recovery seems to appear with age (Dzidic et al. 2018). Whether or not such altered colonization pattern during the first year of life do have a long-term consequences for child’s oral health is, however, not clear. In this study, we found no firm evidence of an elevated prevalence of early childhood caries levels in C-section children. Nevertheless, in contrast to the previous systematic review (Antão et al. 2019), we noticed a weak but statistically significant tendency towards a higher caries prevalence in the C-section group. The certainty of this finding was, however, low due to inconsistencies and a high risk of bias in the majority of the included publications. Notably, even the three studies with moderate and low risk of bias displayed conflicting findings.

Results from meta-analyses of observational studies are in general more hazardous than results from randomised controlled trials. Due to the limited number of studies that fulfilled the inclusion criteria, we combined results from publications with different designs in our analyses, which meant that various methods of sample selection and caries detection were mixed. In, addition, some of the included studies targeted ECC and its risk factors and were, therefore, not designed or power calculated for analyses related to mode of delivery. We calculated unadjusted univariate associations between delivery mode and caries, which was an oversimplification taking the complex aetiology of ECC into account (Kirthiga et al. 2019). Some of the included studies used a blend of confounding factors to adjust the multivariate analyses concerning caries background variables, but we decided to extract the crude dichotomised data to be able to pool the outcome. Reporting a standardized “core outcome set” at a certain age in future projects would certainly facilitate the compilation of data and increase the certainty of evidence in tomorrow’s systematic reviews. Still, the results of present study indicate that the mode of childbirth delivery should be captured and a possible risk factor for ECC.

The authors found high heterogeneity between the included studies, reflecting variations in population size, study group characteristics, outcome age, methods for caries detection and disease rate. The registry-based studies profited from their large sizes, but suffered from weak reliability in the outcome assessment, while a weakness in many cross-sectional trials was the representability of the selected study populations. We are confident that the “exposure” data were reliable although this information in some studies was collected from retrospective questionnaires completed by custodians. A limitation was, however, that all publications lacked detailed information on the reason for the C-section and if it was conducted on maternal request, medical indications or emergencies. Only the registry based study by Brandquist et al. (2017) defined the exposure criteria as “delivery starts by caesarean section” and included both elective and emergency cases. This lack of information may have an impact on the results, since the sociodemographic variables, health beliefs and level of economic development of the societies are important factors and co-variates that can affect both the rate of C-section and the occurrence of caries (Pitts et al. 2017; Jadoon et al. 2019).

The data on the “outcome” were in most studies based on clinical scoring of decayed, extracted and filled teeth on cavity level and this was likely robust enough for the dichotomous outcome measure used here. However, caries is a continuum and cavities takes often years to develop and only one trial included detection of the early, non-cavitated stages of the disease and used bitewing radiographs to detect proximal lesions (Boustedt et al. 2018). This means that the true prevalence of caries was certainly underestimated as the early carious lesions makes up the greater share at all preschool ages, particularly in industrialised countries (André Kramer et al. 2014). The contrasting levels of ECC across the accepted studies should also be noted; the caries prevalence at 3 years of age on cavitated level (deft > 0) was very low (< 9%) in all the Scandinavian studies, while the prevalence was 3–5 times higher in the non-Nordic reports. Also the caries frequency, expressed as mean deft, differed five–tenfold across the publications. These large differences in the event rate, likely mirroring the public dental health structures, access to dental care and community-based preventive measures in the different countries, contribute to the high heterogeneity disclosed here. Another shortcoming was that only two studies reported data from calibration and reliability tests of the examiner(s).

## Conclusion

Within the limitations of this systematic review, we found a weak trend but no firm association between the mode of delivery and the prevalence of early childhood caries. There was an obvious inconsistency and heterogeneity across the included studies but the findings indicate that the mode of delivery should be registered in the paediatric dental records. Further studies based on representative birth cohorts and reporting a standardized core outcome set are warranted to answer the research question with higher certainty.

## Supplementary Information

Below is the link to the electronic supplementary material.

Supplementary file1 (DOCX 15 KB)

Supplementary file2 (DOCX 13 KB)
