# Supplementary material for: Is the mode of childbirth delivery linked to the prevalence of early childhood caries? A systematic review and meta-analysis
Source: Eur Arch Paediatr Dent. 2021 May 10;22(5):765–72. doi: 10.1007/s40368-021-00621-6 (PMC8526466; doi:10.1007/s40368-021-00621-6)
Supplement: Supplementary file 1 — Supplementary file1 (DOCX 15 KB) [file 40368_2021_621_MOESM1_ESM.docx]

**Reference list - excluded publications**

Alshehhi A, Al Halabi M, Hussein I, Salami A, Hassan A, Kowash M. Enamel defects and caries prevalence in preterm children aged 5-10 years in Dubai. Libyan J Med; 2010;15:1, doi: 10.1080/19932820.2019.1705633.

Amin SB, M.D, Karp LM, Benzley LP. Unconjugated hyperbilirubinemia and early childhood caries in a diverse group of neonates. Am J Perinatol. 2010;27:393–7.

Borowska-Strugińska B, Żądzińska E, Bruzda-Zwiech A, Filipińska R, Lubowiecka-Gontarek B, Szydłowska-Walendowska B, Wochna-Sobańska M. Prenatal and familial factors of caries in first permanent molars in schoolchildren living in urban area of Łódź, Poland. Homo. 2016;67:226-34.

Brignardello-Petersen R. Children delivered via cesarean section had a lower risk of developing caries than those delivered vaginally in a low caries prevalence setting. J Am Dent Assoc. 2019;150(6):e96.

Cho GJ, Kim S, Lee HC, Kim HY, Lee K-M, Han SW, Oh M-J. Association between dental caries and adverse pregnancy outcomes. Sci Rep 2020;10,5309 doi:10.1038/s41598-020-62306-2.

Ghazal T, Levy SM, Childers NK, Broffitt B, Cutter GR, Wiener HW, Kempf MC, Warren J, Cavanaugh JE. Factors associated with early childhood caries incidence among high caries-risk children. Community Dent Oral Epidemiol. 2015;43:366-74.

Kuthy RA, Jones M, Kavand G, Momany E, Askelson N, Chi D, Wehby G, Damiano P. Time until first dental caries for young children first seen in Federally Qualified Health Centers: a retrospective cohort study. Community Dent Oral Epidemiol. 2014;42:300-10.

Loureiro L, Bandeira Lopes L, Ventura I. Early childhood caries in five to seven-year-old children, having as variants the mode of delivery and mutans streptococci colonization: a pilot study. Annals Med. 2019;51:sup1, 117. doi: 10.1080/07853890.2018.1562724.

Nasrul N, Hafid F, Ramadhan K, Suza DE, Efendi F. Factors associated with bottle feeding in children aged 0–23 months in Indonesia. Children and Youth Services Review. 2020;116:105251.

Poureslami H, Bafti LS, Hashemi Z, Salari Z. Comparison of occurrence of early childhood caries in two groups of children delivered by cesarean section and normal Birth: A longitudinal study. J Compr Ped. 2012;3:77–81.

Salam R. Non-traditional exposures and childhood dental caries among children 1-5 years old. Temple University, ProQuest Dissertations Publishing, 2017. 10600654.

Sayyed T, Kandil M, Bashir O, Alnaser H. The relationship between term pre-eclampsia and the risk of early childhood caries. J Matern Fetal Neonatal Med. 2014;27:62-5.

Shaker NT. The effects of mode of delivery on Mutans Streptococci colonization and dental caries in a sample of Iraqi children aged 3-5 years from Baghdad city. Mustansiria Dent J. 2017;14:51-7.

Stephen A, Krishnan R, Chalakkal P. The association between cariogenic factors and the occurrence of early childhood caries in children from Salem district, India. J Clin Diagn Res. 2017;11:ZC63-ZC66.

Vandana K, Raju S H, Badepalli RR, Narendrababu J, Reddy C, Sudhir K M. Prevalence and risk-factors of early childhood caries among 2–6-year-old Anganwadi children in Nellore district, Andhra Pradesh, India: A cross-sectional survey. Indian J Dent Res 2018;29:428-33.

Yepes JF, Bush HM, Li HF, Talbert J, Nash DA. Antenatal and intrapartum risk factors for use of emergency and restorative Medicaid dental services for children. Pediatr Dent. 2014;36:405-10.
