# Supplementary material for: Is the mode of childbirth delivery linked to the prevalence of early childhood caries? A systematic review and meta-analysis
Source: Eur Arch Paediatr Dent. 2021 May 10;22(5):765–72. doi: 10.1007/s40368-021-00621-6 (PMC8526466; doi:10.1007/s40368-021-00621-6)
Supplement: Supplementary file 2 — Supplementary file2 (DOCX 13 KB) [file 40368_2021_621_MOESM2_ESM.docx]

**Table S1.** Excluded studies and main reason for exclusion

________________________________________________________________________________

First author, year Reason

________________________________________________________________________________

Alshehhi, 2020 Caries prevalence not reported

Amin, 2010 Children compromised (Unconjugated hyperbilirubinemia)

Borowska-Strugińska, 2016 Out of age, caries reported for permanent teeth only

Brignardello-Petersen, 2019 Secondary publication

Cho, 2020 Caries prevalence not reported

Ghazal, 2015 Caries prevalence not reported

Kuthy, 2014 Caries prevalence not reported

Loureiro, 2019 Caries prevalence not reported

Nasrul, 2020 Caries prevalence not reported

Poureslami, 2012 Caries data not accessible

Salam, 2017 Grey literature, master thesis

Sayyed, 2014 Caries prevalence not reported

Shaker, 2017 Caries prevalence not reported

Stephen, 2017 Caries prevalence not reported

Vandana, 2018 Caries prevalence not reported

Yepes, 2014 Caries prevalence not reported

________________________________________________________________________________
